# Differential expression of pathogenic genes of *Entamoeba histolytica* vs *E*. *dispar* in a model of infection using human liver tissue explants

**DOI:** 10.1371/journal.pone.0181962

**Published:** 2017-08-03

**Authors:** Cecilia Ximénez, Enrique González, Miriam Nieves, Ulises Magaña, Patricia Morán, Marco Gudiño-Zayas, Oswaldo Partida, Eric Hernández, Liliana Rojas-Velázquez, Ma. Carmen García de León, Héctor Maldonado

**Affiliations:** 1 Laboratory of Immunology, Unit of Experimental Medicine, Faculty of Medicine, UNAM, México City, México; 2 Unit of Scientific Vinculation, Faculty of Medicine, UNAM/INMEGEN, México City, México; 3 Sub direction of Pathology, National Institute of Cancerology, México City, México; Universita degli Studi di Parma, ITALY

## Abstract

We sought to establish an *ex vivo* model for examining the interaction of *E*. *histolytica* with human tissue, using precision-cut liver slices (PCLS) from donated organs. *E*. *histolytica-* or *E*. *dispar*-infected PCLS were analyzed at different post-infection times (0, 1, 3, 24 and 48 h) to evaluate the relation between tissue damage and the expression of genes associated with three factors: a) parasite survival (peroxiredoxin, superoxide dismutase and 70 kDa heat shock protein), b) parasite virulence (*EhGal/GalNAc lectin*, amoebapore, cysteine proteases and calreticulin), and c) the host inflammatory response (various cytokines). Unlike *E*. *dispar* (non-pathogenic), *E*. *histolytica* produced some damage to the structure of hepatic parenchyma. Overall, greater expression of virulence genes existed in *E*. *histolytica*-infected versus *E*. *dispar*-infected tissue. Accordingly, there was an increased expression of *EhGal/GalNAc lectin*, *Ehap-a* and *Ehcp-5*, *Ehcp-2*, *ehcp-1* genes with *E*. *histolytica*, and a decreased or lack of expression of *Ehcp-2*, *and Ehap-a* genes with *E*. *dispar*. *E*. *histolytica*-infected tissue also exhibited an elevated expression of genes linked to survival, principally peroxiredoxin, superoxide dismutase and *Ehhsp-70*. Moreover, *E*. *histolytica*-infected tissue showed an overexpression of some genes encoding for pro-inflammatory interleukins (ILs), such as il-8, ifn-γ and tnf-α. Contrarily, *E*. *dispar*-infected tissue displayed higher levels of il-10, the gene for the corresponding anti-inflammatory cytokine. Additionally, other genes were investigated that are important in the host-parasite relationship, including those encoding for the 20 kDa heat shock protein (HSP-20), the AIG-1 protein, and immune dominant variable surface antigen, as well as for proteins apparently involved in mechanisms for the protection of the trophozoites in different environments (e.g., thioredoxin-reductase, oxido-reductase, and 9 hypothetical proteins). Some of the hypothetical proteins evidenced interesting overexpression rates, however we should wait to their characterization. This finding suggest that the present model could be advantageous for exploring the complex interaction between trophozoites and hepatocytes during the development of ALA, particularly in the initial stages of infection.

## Introduction

*Entamoeba histolytica* and *E*. *dispar*, the etiological agents of amoebiasis, infect only human beings and non-human primates. Both species enter the human organism as a cyst in infected water or food. Inside the gastrointestinal tract, the cyst loses its chitin wall and releases eight trophozoites, which proceed to colonize the large intestine or bowel. Whereas 90% of individuals infected with *E*. *histolytica* are asymptomatic, 10% suffer severe invasive tissue damage producing bloody diarrhea (dysentery) and ulcerative lesions, the latter in the form of colitis or amoebic liver abscess (ALA) [1].

On the other hand, there are two *Entamoeba* species: *E*. *dispar* and *E*. *histolytica* are morphologically identical and both can colonize the same ecological niche in the bowel mucosa. However, there are genotypic and phenotypic differences between the two *Entamoeba* species that probably are in large part responsible for the generally more pathogenic nature of *E*. *histolytica*. It has been found that *E*. *histolytica* invades the human host and causes disease, while *E*. *dispar* has usually proven to be noninvasive [2–6]. Nevertheless, important epidemiological evidence now exists of non-pathogenic and pathogenic variants of both *Entamoeba* species [7].

*In vitro* studies have long been employed to examine the interaction of *E*. *histolytica* and/or *E*. *dispar* trophozoites with different biological substrates [8]. *In vitro* models developed for the evaluation of the pathogenic capabilities of distinct strains of *E*. *histolytica* have focused mainly on three virulence factors: amoebapores, the galactose and N-acetyl galactosamine inhibitable lectin (Gal/GalNAc lectin), and cysteine proteases. Additionally, these models have explored the modulation of the host immune response by *E*. *histolytica* during infection (since the parasite needs an inflammatory environment), as well as other mechanisms of parasites to evade the host immune response [9–13].

It has been difficult to develop suitable *in vivo* experimental animal models of *Entamoeba* infection for assessing the pathogenic mechanisms of amoebiasis leading to tissue lesions. Some rodent models have been successfully developed to reproduce damage to hepatic or intestinal tissues, including susceptible strains of mice and resistant strains of gerbils and golden hamsters [14]. Curiously, much of the vast knowledge from *in vitro* models (about the complexity of the host-parasite relationship and the multifactorial origin of pathogenesis) does not correlate with findings from animal models, whether in regard to the bowel or liver [15]. For example, it seems that some *in vitro* effects of virulence factors described for *Entamoeba* species are not manifested with*in vivo* experimental models of infection [14–16].

Another model has been developed that offers some of the advantages of both the *in vitro* and *in vivo* models. This is an *ex vivo* model using precision-cut liver slices (PCLS) [17] from live hepatic tissue. Having a specific diameter and identical thickness, these tissue slices are placed in culture microplates and can survive under controlled conditions for enough time to allow for the observation of infection. The PCLS of this *ex vivo* model have virtually all the characteristic cells of the liver parenchyma, as well as all the components of the organ of origin, which allows for the interaction of trophozoites with the epithelial cells and proteins of the extracellular matrix [18]. Since the tissue slices preserve the architecture and functionality of the organ, metabolic processes can be analyzed. Another advantage of PCLS is the reduction in the number of animals required, which is much greater for *in vivo* studies [19].

Despite the multiple applications given to PCLS, their use for examining the mechanisms of *E*. *histolytica* infection in hamster liver was reported for the first time by Carranza-Rosales *et al*. in 2010 [20, 21]. The process of infection described therein is comparable to the observations made with the hamster ALA model. On the other hand, Bansal *et al*. employed an *ex vivo* model in 2009toevaluate invasive intestinal amoebiasis inhuman colon explants [22] by exploring the host-parasite interaction (including the human immune response) during the initial stages of host tissue infection. The possibility of utilizing the PCLS model with human tissue creates an excellent alternative for better understanding the complex host-parasite interaction during the *E*. *histolytica* infection.

Multiple molecules have been detected and analyzed in the pathogenicity of *E*. *histolytica*, and it is known that some others exist but have not yet been found. As aforementioned, the three well-studied virulence factors of these amoebas are amoebapores, the adhesion molecule Gal/GalNAc lectin, and cysteine proteases.

The Gal/GalNAc-lectin (*Eh*lect) is involved in the earliest events of trophozoite adherence to the mucosa and epithelial cells of the intestine [23, 24]. These initial interactions can be inhibited by monoclonal antibodies directed against the recognition domain of carbohydrates in the Hgl subunit [25]. It is also known that this lectin participates in the processes of resistance of *E*. *histolytica* against the human complement. Accordingly, the lectin binds to C8 and C9 components of serum complement and prevents the formation of the membrane attack complex (C5b-9) [26].

The amoebapores of *E*. *histolytica* (*Eh*amp) forma family of small proteins. They are classic pore-forming proteins, functionally and structurally like the granulolysin proteins in NK cells and cytotoxic lymphocyte granules [9]. The amoebapore inserts itself into the cell membranes and subsequently produces oligomers through peptide-peptide ionic channel interactions, which generally prompt cytolysis of host white blood cells [27, 28]. A peptide homologous to this molecule in *E*. *histolytica* has been identified in *E*. *dispar*. It is located in cytoplasm granules and has a 95% identity in its primary structure with the molecule present in the *E*. *histolytica* species. Regarding functional properties, the amoebapore of these two *Entamoeba* species are also similar, showing increased activity at acid pH. Despite these similarities, amoebapore activity is 60% lower in *E*. *dispar* than *E*. *histolytica*, possibly due to the presence of a shorter alpha helix amino-terminal region of the amoebapore in the former species[27]. Although there have been many advances in the biochemical and molecular characterization of amoebapores, the effect of amoebapores on the pathogenic behavior of *E*. *histolytica* is not fully understood. Moreover, the *in vitro* inhibition of amoebapore activity has not yet been achieved [29, 30].

The other essential virulence factor in the pathogenesis of *Entamoeba* is its secretion of cysteine proteases (*Eh*CP),which digests proteins of the extracellular matrix, enabling trophozoites to penetrate deeper into the tissue of intestinal submucosa and disperse this layer [31, 32]. Through *in vitro* assays, digestion of purified proteins (collagen, elastin, fibrinogen and laminin) has been measured and the results compared between *E*. *histolytica* and *E*. *dispar*, as well as between strains of different virulence [33].

*E*. *histolytica* secretes 10–1000 times more of these molecules than *E*. *dispar* [33]. Hybridization assays have demonstrated the existence of *E*. *histolytica* genes encoding for two cysteine proteases, *Ehcp-1* and *Ehcp-5*, which are not found in *E*. *dispar*. In the latter species, only six genes encode for cysteine proteases, and only two of these are expressed. This might explain the low levels of cysteine proteinase activity in *E*. *dispar*, which in turn could be related to its characteristic non-pathogenicity [34, 35].

By utilizing *in vivo* rodent models of ALA, the importance of cysteine proteinases in the virulence of *E*. *histolytica* has been clearly established. For instance, the treatment of these trophozoites with a specific inhibitor of cysteine proteases (E-64) can prevent the formation or reduce the size of liver abscess in SCID mice [36]. Likewise, the overexpression of the *Ehcp-5* gene in transfected trophozoites results in larger abscesses in the gerbil model [37].

The role of calreticulin (CRT) in the pathogenicity of *E*. *histolytica* species has been studied over the past decade. This multifunctional protein, associated with the endoplasmic reticulum (ER), can be found in all eukaryotic cells and is highly conserved across a wide range of species [38]. Although CRT was first detected primarily as a resident of the ER lumen, it has since been identified in a wide variety of cell compartments [39]. Due to such ubiquitousness, it participates in multiple functions of eukaryotic cells [40, 41]. Calreticulin has been observed extracellularly in the saliva of mosquitoes and ticks [42, 43], in the blood and serum of different mammals (including humans), and in the extracellular space of different cell types stimulated *in vitro* [44].

CRT is one of the most immunogenic molecules of *E*. *histolytica*, known to induce a strong antibody immune response in humans [45]. Recently, our group reported the cloning of the *Ehcrt* gene and the expression of a recombinant protein. The *in vivo* expression of this protein was demonstrated for the first time in a hamster model of ALA, in which the expression of the *Ehcrt* gene increased during the initial stages of abscess development. This finding suggests that CRT can be directly or indirectly involved in the pathogenic mechanisms of *E*. *histolytica* [46], likely forming part of the parasite mechanisms aimed at evading the host immune response (as shown for other protozoa, *Trypanosoma cruzi* and *Schistosoma*) [47, 48].

There was a recent report on the ability of *T*. *cruzi* CRT to specifically bind to the C1q component of the classic pathway of serum complement in an infected host, thus inhibiting the activation of this immune response amplification system [47].The same mechanism has also been described for *E*. *histolytica* trophozoites [49]. Additionally, the interaction between C1q and trophozoites previously stimulated with Jurkat cells leads to a C1q-CRT interaction on the membrane of the trophozoites [50].

Trophozoites entering the blood flow encounter molecules of the host innate immune response (e.g., the serum complement activated through alternative pathways), cytotoxic compounds, and higher oxygen tensions than those usually found in the gut. Moreover, trophozoites are exposed to other efficient host defense mechanisms such as reactive oxygen and nitrogen intermediates produced by phagocytic cells. These compounds are extremely toxic and are secreted in copious amounts in infected tissue.

It has been established experimentally that trophozoites invading the liver are very sensitive to blood complement [51,52]. Several strategies have been developed by prokaryotic and eukaryotic organisms to protect themselves against oxygen toxicity, employing enzymes that destroyperoxides and superoxide anions, as well as small molecules as antioxidants (e.g., vitamins E and C), and thiol groups as scavengers of transient free radicals. They possess several enzymes to defend from oxidative stress, such as peroxiredoxin (Prx), superoxide dismutase, flavoprotein A, ferredoxin, thioredoxin (Trx), and Trx reductase, capable of catalyzing the conversion of superoxide to O_2_ and hydrogen peroxide (H_2_O_2_) [53,54], and a flavin reductase (NADPH: flavin oxidoreductase) also able to reduce O_2_ to H_2_O_2_ [55].

Proteins are major targets for oxidants because of their abundance in biological systems and their high rate constants for reaction during several types of stresses, chaperons increase folding and prevent protein misfolding/aggregation and at the same time promote degradation of oxidized proteins by the lysosome/vacuolar system. Heat shock proteins (Hsp) are ATP-dependent enzymes usually classified according to their molecular weights (Hsp40, Hsp60, Hsp70, Hsp90, Hsp100 and the so-called small Hsps). Several HSPs have been reported in *E*. *histolytica*: Hsp10, Hsp40, Hsp60, Hsp70, Hsp90, Hsp100 and Hsp101. Except for Hsp10 and Hsp60, most of them (transcripts or proteins) are overexpressed by heat, high concentrations of; O_2_,H_2_O_2_ and NO[52].

The aim of the present study was to evaluate, for the first time, the accessibility and reproducibility of an *E*. *histolytica* infection by using the *ex vivo* PCLS model with human liver tissue. We herein demonstrate that this approach facilitates the exploration of both aspects of the host-parasite relationship: the survival/virulence behavior of the parasite as well as the immune response in host tissue. *E*. *dispar*-infected specimens were included as a reference control, representing a non-pathogenic *Entamoeba* infection. The expression of genes linked to the host inflammatory response was assessed as well.

## Materials and methods

### Ethics statement

The protocol of the present study was previously authorized by the Ethics in Research Committee of Faculty of Medicine in the National Autonomous University of Mexico (UNAM). Tissue samples were obtained after the signature of informed consent to authorize the autopsy and donation of the liver specimen for experimental purposes. All the procedures took place at the Department of Pathology and Postmortem Service of the General Hospital “Dr. Eduardo Liceaga” of Mexico City, belonging to the Health Ministry.

### Amoebic cultures

Trophozoites of the pathogenic *E*. *histolytica* HM1: IMSS and the non-pathogenic *E*. *dispar* SAW760 were grown under axenic conditions with TYI-S33 or TYI-S2, respectively [56]. All trophozoites were harvested at the logarithmic phase of growth (after 48h). The *E*. *histolytica* trophozoites were passed bimonthly through hamster liver to maintain virulence [57].

### Preparation of precision-cut liver slices (PCLS)

A fragment of 2 x 3 cm of the upper right lobe of the liver was obtained from individuals 4–8 h postmortem. In no case did the cause of death compromise liver integrity. The specimen was immediately introduced in KREBS medium for transportation to the laboratory. A specimen of liver tissue of 8.0 mm in diameter was punched out and introduced in 5% agar at 37°C in an atmosphere of O_2_-CO_2_ (95:5). This core of liver tissue was then sliced in the presence of oxygenated KB buffer (4°C, 95:5 O_2_-CO_2_) by using a vibratome (VT1000S, Leica) to obtain 300 μm PCLS, as previously described [17]. Liver slices were gently placed into each well (with 1 ml of DMEM/F12 medium, Invitrogen) of 24-well polystyrene microplates and subsequently pre-incubated for 1 h at 37°C in a cell culture incubator to stabilize the tissue.

### *Ex vivo* infection

The PCLS were infected with an inoculum of 1X10^5^ trophozoites of *E*. *histolytica* or *E*. *dispar* and suspended in 1 ml of culture medium DMEM/F12. They were then mixed (1:1) with TYI-S33 or TYI-S2, respectively, and supplemented with 2.24 g/L of sodium bicarbonate, 50 μg/ml gentamycin, 25 mM glucose, and 1% of a mixture of insulin-transferrin selenium mix (ITS) (Sigma Chemicals Co.). The tissue was incubated at 37°C in a humid atmosphere of 95:5 of O_2_: CO2 for 2 h to allow for the adhesion and penetration of the trophozoites.

Following this period of early interaction, incubation continued under the same conditions in the microtiter plates, but now with agitation (30 to 50 rpm). After different intervals of infection (0, 1, 3, 24 and 48 h), the tissue was processed for histopathological, immuno-histochemical and molecular assays. Each experiment was performed in duplicate. PCLS without *E*. *histolytica* or *E*. *dispar* infection were included as the control.

### Histopathological and immuno-histochemical assays

After the corresponding incubation period, the tissue slices were fixed in 10% formaldehyde and embedded in paraffin to obtain cuts of 5 μm. These were later processed and stained with standard PAS reagent and examined by light microscopy to identify the parasite and evaluate histological structure.

To analyze the interaction between host cells and some amoebic proteins directly involved in certain processes of amoebic pathogenicity, we tested the lectin from 170 kDa and CRT. These were detected *in situ* within the trophozoites present in the PCLS by utilizing immuno-staining with specific antibodies produced in rabbit (anti-*Eh*lect, kindly donated by Dr. Rosario López-Vancell) [11] and mice (anti-*Eh*CRT) [46]. The slices of biopsies infected for different amounts of time were de-paraffinized and then treated to recover the antigen by heating at 95°C for 5 min in a solution of citrates at 10 mM and pH 6.5.

The slices were blocked with 3% PBS-BSA solution and 1% SB for 12 h at 4°C, and incubated with 1:100 dilutions of the different antibodies for 12 h at 4°C. A secondary antibody diluted 1:1000 (goat anti-rabbit IgG or goat anti mouse IgG coupled to alkaline phosphatase; Sigma) was employed to reveal the Ag-Ab reaction. The NBT/BCIP solution (Sigma) served as substrate of the enzyme. Finally, the slices were counterstained with aqueous eosin and mounted using gelatin/agarose (Sigma) for light microscopy analysis.

All photomicrographs were obtained using a Hyper HAD Color Video Camera (Model SSC—DC30; Sony Corporation, Japan). The following method of semi-quantification was used for immuno-histochemical detection of Ehcrt, and Ehlect. After acquisition of the images using the digital camera, the experimental image files were processed using the PhotoImpact software (Ulead PhotoImpact SE version 3.02; Ulead Systems, USA). To obtain the better and homogeneous signals, and then selected for analysis of relevant regions. These selected regions were then digitally analyzed using the Image-ProPlus Analysis Software (version 4.5.0.19, Media Cybernetics, Inc., USA).

### cDNA obtained in situ from *E*. *histolytica*- or *E*. *dispar*-infected PCLS

RNA was obtained and converted to cDNA with RT-PCR *in situ*, utilizing a modified version of a previously reported method [58–60]. Briefly, after infection for 0, 1, 3, 24 or 48 h, each PCLS was treated with proteinase K (0.5 μg/ml; Sigma) for 30 min at room temperature and subsequently with DNase1 (1U/sample) for 48 h at room temperature. PCLS were then washed using water treated with DPC. The reverse transcriptase reaction was performed with the RT-Super Script II enzyme (Invitrogen), following the indications of the supplier.

Briefly, 25 μl DPC, treated water containing 2.5 μl of oligo (dT), 10mM dNTP mix, 5 μl 5X First-Strand buffer, 1.2 μl 0.1 M DTT, 0.25 μl of recombinant ribonuclease inhibitor (40U/μl), and reverse transcriptase (100U/section) were added to each slice and were incubated at 42°C for 2 h in a moist environment. Thereafter, synthesized cDNA was recovered and quantified by spectrophotometry at 260/280 nm, to await use at 15ng/μl in the qPCR assays.

### Expression of amoebic genes (related to pathogenicity) and host genes (of the immune response)

For the distinct times of exposure to infection, the expression of *Entamoeba* genes associated with pathogenicity and human genes coding for ILs were evaluated by real time amplification polymerase chain reaction (qPCR). The specific oligonucleotides for the genes studied are shown in Table 1. The cDNAs previously obtained from the *in-situ* RT-assays served as a template for the reactions of qPCR by using the Step One-Applied Biosystems and the Quantitec SYBER Green PCR reaction kit (Qiagen). Amplification was carried out with 60 cycles in 3 stages, including denaturing at 95°C for 10 sec, alignment at 57°C for 30 sec, and elongation at 72°C for 10 sec. Finally, the dissociation curve was constructed. The amplification of each gene was performed in triplicate and its differential expression calculated through normalization against a reference gene (the *Ehα*-actin gene of the trophozoites and the human β-actin of the ILs). PCLS of human liver tissue without trophozoites served as the control. Differences in gene expression levels were compared between *E*. *histolytica*- and *E*. *dispar*-infected tissue. The data were analyzed with the 2-ΔΔCT method described by Livak and Schmittgen (2008) [61] and the validation method reported by Yalcin (2004) [62].

**Table 1:** Primers and their sequences used in this study.

Gene	Access number	Sise of products	Forward primer (5´- 3´)	Reverse primer (5´- 3´)
			***Genes of Homo sapiens sapiens***	
tnf-α	X02910.1	107	GCCCTACTATTCAGTGGCGA	GAGCTTCTTCCCACCCACAA
ifn-α	NM_024013	182	CTTGTGCCTGGGAGGTTGTC	TAGCAGGGGTGAGAGTCTTTG
il-4	NM_000589	194	GTGCACCGAGTTGACCGTAA	TGTCGAGCCGTTTCAGGAAT
il-8	NM_000584.3	183	GGTGCAGTTTTGCCAAGGAG	TTCCTTGGGGTCCAGACAGA
il-10	NM_000572.2	135	AAGACCCAGACATCAAGGCG	AGGCATTCTTCACCTGCTCC
il-17	NM_002190.2	133	CCTTGGAATCTCCACCGCAA	GCTGGATGGGGACAGAGTTC
tnf-β	X01393.1	155	TCTCCCCATTCTGCCTCCATT	GGATGGTTCAGGGAGTGTGGG
Gen of reference *Hsactin*-β	NM_001101.3	166	CTCACCATGGATGATGATATC	AGGAATCCTTCTGACCCATGC
			***Genes of E*. *histolytica/E*. *dispar***	
*Ehcrt*	XM_650149.1	355	TGGACCAGATGTATGTGGAGG	TGGTGCTTCCCATTCTCCATC
*Ehamp*-a	X70851.1	212	AAGGAGAAATCCTCTGCAAC	CAAATAGCATTGGCATCAAC
*Ehcp*-5	XM_645845.2	255	GTTGCCGCTGCTATTGATGC	ACCCCAACTGGATAAGCAGC
*Ehcp*-1 *Ehcp*-2	XM_645064.2 XM_645550	245 115	CATGTAGAAGTGATGTGAAAG ATCCAAGCACCAGAATCAGT	TTCTTTCCCATCAACAACAC TTCCTTCAAGAGCTGCAAGT
*Ehlect*	AF337950.1	281	ACCAGTGAATGGAGCATGTGT	TTG TGC ATT CGC CTT CTC CT
*Ehprd*	XM_646911.2	121	TCAAGAGAAAGAATGTTGTTGT	ACATGGACAATATGCTGCTGC
*Ehsod*	X70852.1	172	GCAGCCCAAGCATGGAATCA	ACCAACACCATCCACTTCCA
*Ehhsp*-70	XM_001734367.1	135	GAAACAGAACCACGTCCAGTT	TTACGTCCTCCAAGTCTCCAAT
Gen of reference *Ehactin*-α	XM_650064.2	211	AGCTGTTCTTTCATTATATGC	TTCTCTTTCAGCAGTAGTGGT

### Identification of genes related to pathogenicity

Based on the sequencing and annotation of the *E*. *histolytica* genome [63], microarray and NGS approaches have been developed to examine gene expression in this organism. The data obtained revealed important regulatory networks involved in strain phenotype differences, colonic and hepatic invasion, and responses to stress [64–69].

We select 20 genes to assess their differential expression (S1 Table) the sequences were analyzed and primers for qPCR assays were designed. These primers were used in the qPCR performed in the PCLS samples.

### Statistical analysis

The results are expressed as the mean ± the standard deviation of at least two duplicates of each condition for 5 independent samples. The statistical analysis was performed with GraphPad Prism 5 software. The Student’s *t* test was used to compare differences between PCLS infected with *E*. *histolytica* versus *E*. *dispar*. Differences were considered statistically significant when the p-value was <0.05.

## Results

### Pathological study and immuno-histochemical assays

Liver tissues were obtained from 11 donors, 8 males and 3 females ranging in age from 18 to 74 years. The average postmortem time for obtaining the liver was 7 h. The donors had different causes of death (Table 2), which in no case compromised the liver.There was no correlation between the gender or age of the donors regarding the susceptibility of the PCLS to develop an invasive process (data not shown).

**Table 2:** Summary of characteristics for donors of human livers.

Case	Gender	Age	Time postmortem (h)	Cause of death
1	Male	73	6	Severe aortic stenosis, brain stroke
2	Female	28	4	Kidney failure
3	Male	66	7	Pneumonia, hypertension
4	male	18	6	Kidney failure
5	Female	50	8	Gastric carcinomatous
6	Male	56	8	Cardiomyopathy
7	Male	60	9	Brain edema, DMT2, hypertension
8	Male	68	8	Kidney failure
9	Male	48	8	Respiratory failure (EPOC)
10	Male	74	8	DMT2, nephropathy
11	Female	49	7	Pneumonia, respiratory failure (EPOC)

DMT2: Diabetes mellitus type 2

**Fig 1:**
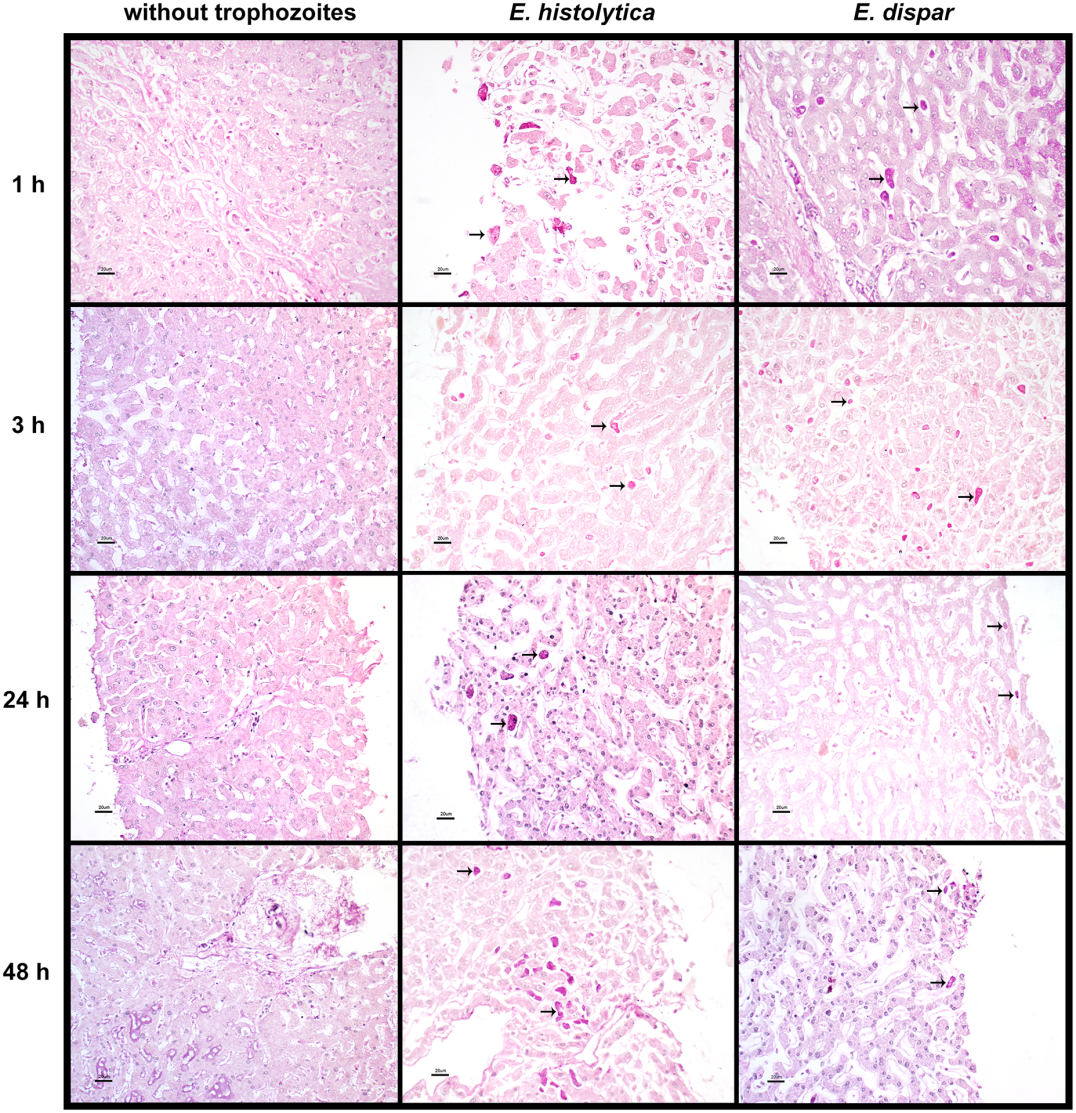
Histological analysis of the presence of trophozoites. PCLS from human liver tissue were infected with *E*. *histolytica* or *E*. *dispar* trophozoites at different times (0, 1, 3, 24 and 48 h). The column entitled “without trophozoites” corresponds to the control. Tissues were stained with PAS. The arrows point out some illustrative trophozoites in representative images. Scale bar = 20 μm.

Whereas *E*. *dispar*-infected tissue was undisturbed, *E*. *histolytica*-infected tissue was damaged. Meanwhile, the uninfected PCLS (Fig 1, without trophozoites) displayed normal morphology throughout the experiment (0–48 h).

The histopathological study evidences trophozoites located mainly on the periphery of the sinusoids during the first few hours. At 3h, some intravascular trophozoites were detected. At 24 h of interaction, 12.5% of the initial number of trophozoites were attached to epithelial cells and 4% to hepatocytes with some degree of degeneration, while 6% were located intravascularly.

Most of these amoebas were located on the periphery of hepatic tissue, in areas showing slight sinusoidal dilatation. Despite this dilatation of the sinusoids, we did not observe a classic inflammatory process. Inflammation was evident only on the periphery of the PCLS, which could point to a defective cut of hepatic tissue. Although, there were some immunologic cells (e.g., lymphocytes and histiocytes), it cannot be concluded that the presence of the trophozoites per se is the only cause of the damage herein manifested. On the other hand, the presence of bi-nucleated hepatocytes indicated the existence of tissue regeneration.

**Fig 2:**
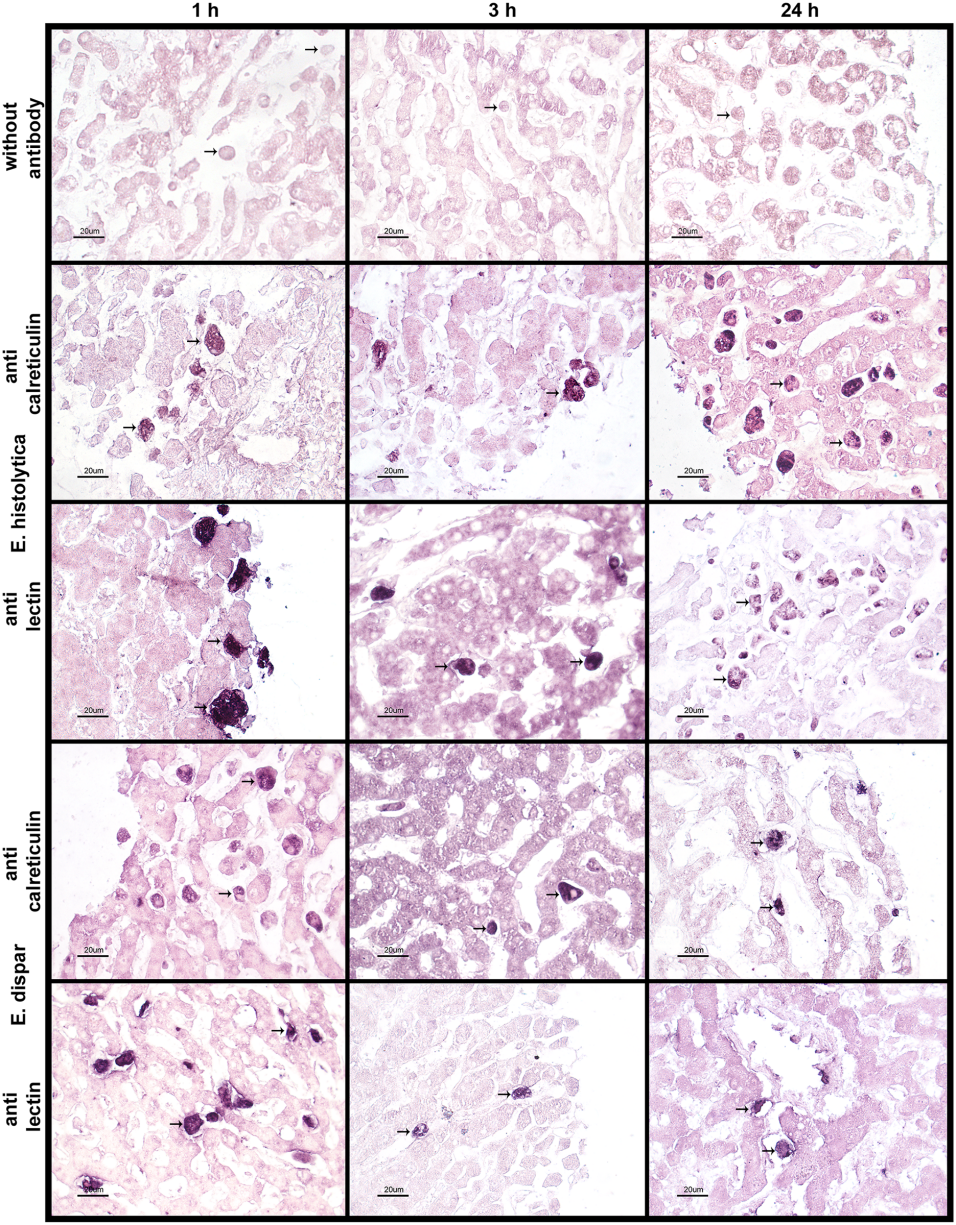
Immuno-histochemical detection of EhCRT and Ehlect. PCLS from human liver tissue were inoculated *ex vivo* with *E*. *histolytica* or *E*. *dispar* trophozoites at different times (1, 3, 24 and 48 h). Tissues were counterstained with eosin. The arrows point out some illustrative trophozoites in representative images. Scale bar = 20 μm.

Examination was made of the immunolocalization of *Eh*CRT and *Eh*Gal/GalNAc lectin in the PCLS incubated with trophozoites of *E*. *histolytica* or *E*. *dispar* for different times (Fig 2). Densitometric analysis identified differences in the expression of these proteins over the course of the experiments (S1 Fig). These data agree with the results of the qPCR assays.

### Expression of genes associated related to pathogenicity in the ex vivo model

The expression of pathogenic genes was compared between *E*. *histolytica* or *E*. *dispar* infected PCLS, all they are shown in Table 3. Levels of *EhGal/GalNAc* lectin were higher from 24 to 48h (p = 0.04), mainly in the *E*. *histolytica*-infected PCLS.

**Table 3:** Relative quantification of genes *E*. *histolytica* or *E*. *dispar* associated with pathogenicity and parasite survival.

CLAVE	Time (h)	RQ ± SD *E*. *histolytica*	RQ ± SD *E*. *dispar*	p value
*Eh*crt	1	9.93 ± 4.6	2.8 ± 1.2	0.11
	3	**10.6 ± 6.5**	**2.1 ± 1.5**	**0.047**
	24	4.2 ± 3.15	0.35 ± 0.03	0.45
	48	2.17 ± 0.55	0.87 ± 0.32	0.78
*Eh*amp	1	**13.22 ± 6.3**	**1.1 ± 0.6**	**0.05**
	3	**3.3 ± 1.4**	**0.2 ± 0.1**	**0.046**
	24	0.35 ± 0.2	0.62 ± 0.2	0.082
	48	0.49 ± 0.3	1.3e-4 ± 0.7e-5	0.43
*Eh*lect	1	0.51 ± 0.22	1.2 ± 0.1	0.12
	3	1.73 ± 0.83	3.9 ± 1.3	0.430
	24	**16.2 ± 8.1**	**0.8 ± 0.3**	**0.042**
	48	**6.1 ± 1.4**	**0.22 ±0.1**	**0.041**
*Eh*cp1	1	**5.02 ± 2.1**	**0.38 ± 0.11**	**0.02**
	3	**5.4 ± 0.43**	**0.48 ± 0.18**	**0.034**
	24	1.37 ± 0.6	0.67 ± 0.2	0.16
	48	0.52 ± 0.34	0.56 ± 0.35	0.35
*Eh*cp2	1	**7.1 ± 0.9**	**4.2 ± 0.3**	**0.05**
	3	1.8 ± 0.3	0.9 ± 0.4	0.24
	24	1.23 ± 0.31	0.8 ± 0.4	0.53
	48	1.11 ± 0.1	0.9 ± 0.3	1.13
*Eh*cp5	1	**3.5 ± 0.9**	**1.6 ± 0.21**	**0.022**
	3	**5.22 ± 0.5**	**0.3 ± 0.2**	**0.026**
	24	**13.3 ± 6.1**	**0.65 ± 0.2**	**0.017**
	48	1.44 ± 0.6	0.83 ± 0.3	0.157
*Eh*hsp70	1	1.82 ± 0.7	2.8 ± 0.7	0.52
	3	7.81 ± 0.8	6.8 ± 3.3	0.39
	24	**18.9 ± 0.5**	**0.7 ± 0.3**	**0.04**
	48	**2.24 ± 0.7**	**0.24 ± 0.05**	**0.012**
*Eh*prd	1	12.6 ± 5.8	6.1 ± 2.3	1.05
	3	**7.1 ± 2.46**	**2.5 ± 0.8**	**0.043**
	24	**4.35 ± 1.3**	**0.43 ± 0.24**	**0.05**
	48	1.9 ± 0.7	0.53 ± 0.3	0.26
*Eh*sod	1	0.88 ± 0.34	0.84 ± 0.4	1.5
	3	**6.3 ± 1.4**	**3.1 ± 1.2**	**0.032**
	24	**2.5 ± 1.1**	**0.81 ± 0.22**	**0.046**
	48	0.72 ± 0.4	0.32 ± 0.2	0.85

The expression of different genes in PCLS infected with *E*. *histolytica* or *E*. *dispar* trophozoites for different time spans (1, 3, 24 and 48 h) was determined by qPCR. The genes associated with pathogenesis were *Ehcrt*, *Ehcp-1*, *Ehcp*-2 *Ehcp-5*, *EhLgl* and *Ehamp-a*. The genes related to oxidative or thermal stress were *Ehprd*, *Ehsod* and *Ehhsp-70*. (RQ) relative quantification, (SD) standard deviation, data expressed as the mean of 5 separate assays. p value calculated by Student t-test.

*Ehamp-a* was overexpressed only for the first 3h of interaction (1h, p = 0.05; 3h, p = 0.04). Thereafter, the expression of this gene decreased in both species. The differences between the expression of genes in *E*. *histolytica* or *E*. *dispar-infected PCLS* were statistically significant.

Two protease genes (*Ehcp*-1 and *Ehcp*-5) were expressed in *E*. *histolytica* amoebas in infected PCLS that were not present in the *E*. *dispar* genome. Cysteine protease *Ehcp*-5 was overexpressed from 1 to 24 hof cell to cell interaction (1 h, p = 0.02; 3 h, p = 0.02; 24 h, p = 0.017). *Ehcp-1* was overexpressed only during the first 3 h of infection (1 h,p = 0.02; 3h, p = 0.034), thereafter level decreased up to24h post-infection.On the other hand, the *Ehcp*-2 gene was detected in both species. Whereas the expression of *Ehcp*-2 increased steadily in *E*. *histolytica* until the end of 24h, in *E*. *dispar* it was only overexpressed during the first hour of infection(p = 0.05)

Regarding the *Ehcrt* gene, we observed overexpression for the first 3 h, followed by reduced expression in both species (1h, p = 0.1; 3h, p = 0.04). Despite the similar pattern of behavior, the expression of this gene was higher in PCLS infected with *E*. *histolytica* than in PCLS infected with *E*. *dispar*.

Likewise, there were differences between the pathogenic and non-pathogenic species in relation to the levels or in the timing of expression of *Ehsod*, *Ehprd* and *Ehhsp*-70 (Genes associated with processes of protection of the parasite to hostile environmental changes) (Table 3). For example, *Ehprd* and *Ehsod* were overexpressed during the first three hours of PCLS-trophozoite interaction. Although *Ehprd* steadily decreased, the overexpression of *Eh*sod dropped sharply. The level of expression of these genes was higher in *E*. *histolytica* than in *E*. *dispar*. Difference between these two species were detected in the expression of *Ehprd* at 3h (p = 0.043) and at 24h (p = 0.05). A similar pattern of differences was found for the expression of *Ehsod* that showed a significant overexpression between 3 and 24 h. On the other hand, expression of *Ehhsp-70* increased in both *Entamoeba* species, although, during the first 3 h of infection there were not statistical differences. In the last24 to 48 h of PCLS-trophozoite interaction with *E*. *histolytica* we did observe statistical significant differences (24 h p = 0.04, 48h p = 0.01).

### Post-infection expression of genes associated with the immune response

**Fig 3:**
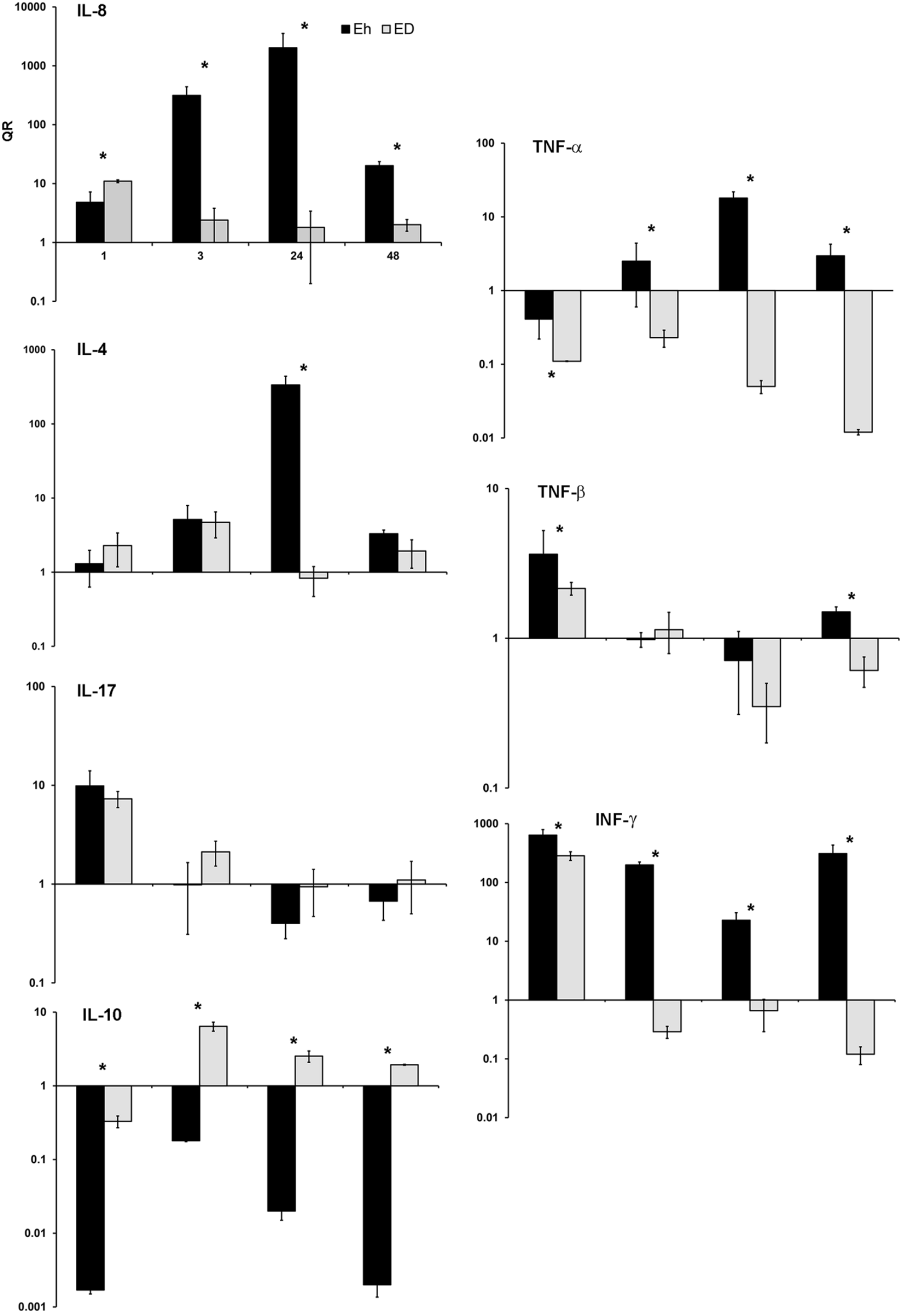
Expression levels of genes linked to the immune response in human liver tissue. After human PCLS were *ex vivo* infected with *E*. *histolytica* or *E*. *dispar* trophozoites at different times (1, 3, 24 and 48 h), the relative quantification (RQ) was determined (by qPCR) for the mRNA of some genes encoding for human cytokines. RQ represents a logarithmic scale, expressed as the mean of separate assays. *p<0.05.

The innate immune response was also evaluated presently in *E*. *histolytica-* and *E*. *dispar*-infected PCLS. The gene expression of different cytokines, determined by qPCR, was different during the infection with each of these two species. The relative quantification (RQ) levels of some interleukin (il) genes are shown in (Fig 3). The *il*-8 gene was overexpressed during the first 24 h of the PCLS-*E*. *histolytica* interaction, followed by a sustained decrease inexpression until the end of the assay (48 h). In the *E*. *dispar*-infected PCLS, the overexpression of this gene was greater, but lasted only a brief time (the first 60 minutes). The difference in the expression level of the *il*-8 gene between *E*. *histolytica*-and *E*. *dispar*-infected PCLS was statistically significant (p = 0.03).

The *tnf*-α gene was overexpressed in *E*. *histolytica*-infected PCLS, but was not expressed at all in *E*. *dispar*-infected tissue (Fig 3). Its overexpression in the former species increased continuously from 3 to 24 h of cell-to-cell interaction, and then steadily diminished up to the end of the assay (48 h).

Regarding the *tnf*-β gene, an elevated expression was exhibited only for the first hour post-infection in both *E*. *histolytica*- and *E*.*dispar*-infected PCLS (p = 0.04). However, the level of expression was higher for the *E*. *histolytica* infection (p = 0.03) (Fig 3).

In the case of the *il*-4 and *il*-17 genes, an elevated expression was found for *E*. *histolytica-* or *E*. *dispar*-infected PCLS. For *il*-4, this increase was only statistically significant at 24 h post-infection (p = 0.02; Fig 3). For *il*-17, the overexpression was detected during the first hour after infection with each of the *Entamoeba* species. Differences in expression between *E*. *histolytica-* and *E dispar*-infected PCLS were not statistically significant(p = 0.06) (Fig 3).

The *inf*-γ gene showed elevated levels of expression, especially with *E*. *histolytica* infection. Its overexpression after *E*. *dispar* infection lasted only 1 h, followed by a rapid drop to a low level that lasted until the end of experiment (Fig 3).

The expression of the *il*-10 gene increased in the presence of *E*. *dispar* trophozoites for the first 3 hrs., then decreased to a minimum level until the end of the assay. During the *E*. *histolytica* infection, contrarily, this gene was always underexpresed (Fig 3).

### Identification of others parasite genes associated with pathogenicity

From the 20 selected genes from literature, only 16 genes due amplify, results are shown in Table 4, the other 4 genes do not amplified with our primers, and in our conditions. It was possible to identify some genes on both *Entamoeba* species that could involve in pathogenicity (Table 4), for example *Eh*20 kDa antigen, *Eh* peptidase, immune dominant variable surface antigen (*Eh*dovasa)and the AIG-1 protein, and others additional genes that appear to be involved in mechanisms for the protection of the trophozoites in different environments, such as the gene for 20 kDa heat shock protein (*Eh*hsp20) and other genes including those for thioredoxin-reductase (*Eh*thiored) and oxide reductase (*Eh*oxired). Nine hypothetical protein genes were detected (Table 4,) some of them were overexpressed in the PCLS infected with *E*. *histolytica* (*Eh*hypo1 and *Eh*hypo8) and the *Eh*hypo3 which only was expressed in *E*. *dispar* PCLS infection. Although, the function of these hypothetical genes is unknown, it is possible that in *E*. *histolytica* they encode for proteins linked to pathogenicity.

**Table 4:** Expression levels of genes linked to mechanisms of pathogenesis in the *ex vivo* infection model, selected by bibliographic revision.

Clave	Time (h)	RQ ± SD *E*. *histolytica*	RQ ± SD *E*. *dispar*	p value
*Eh*crt	1	9.93 ± 4.6	2.8 ± 1.2	0.11
	3	**10.6 ± 6.5**	**2.1 ± 1.5**	**0.047**
	24	4.2 ± 3.15	0.35 ± 0.03	0.45
	48	2.17 ± 0.55	0.87 ± 0.32	0.78
*Eh*amp	1	**13.22 ± 6.3**	**1.1 ± 0.6**	**0.05**
	3	**3.3 ± 1.4**	**0.2 ± 0.1**	**0.046**
	24	0.35 ± 0.2	0.62 ± 0.2	0.082
	48	0.49 ± 0.3	1.3e-4 ± 0.7e-5	0.43
*Eh*lect	1	0.51 ± 0.22	1.2 ± 0.1	0.12
	3	1.73 ± 0.83	3.9 ± 1.3	0.430
	24	**16.2 ± 8.1**	**0.8 ± 0.3**	**0.042**
	48	**6.1 ± 1.4**	**0.22 ±0.1**	**0.041**
*Eh*cp1	1	**5.02 ± 2.1**	**0.38 ± 0.11**	**0.02**
	3	**5.4 ± 0.43**	**0.48 ± 0.18**	**0.034**
	24	1.37 ± 0.6	0.67 ± 0.2	0.16
	48	0.52 ± 0.34	0.56 ± 0.35	0.35
*Eh*cp2	1	**7.1 ± 0.9**	**4.2 ± 0.3**	**0.05**
	3	1.8 ± 0.3	0.9 ± 0.4	0.24
	24	1.23 ± 0.31	0.8 ± 0.4	0.53
	48	1.11 ± 0.1	0.9 ± 0.3	1.13
*Eh*cp5	1	**3.5 ± 0.9**	**1.6 ± 0.21**	**0.022**
	3	**5.22 ± 0.5**	**0.3 ± 0.2**	**0.026**
	24	**13.3 ± 6.1**	**0.65 ± 0.2**	**0.017**
	48	1.44 ± 0.6	0.83 ± 0.3	0.157
*Eh*hsp70	1	1.82 ± 0.7	2.8 ± 0.7	0.52
	3	7.81 ± 0.8	6.8 ± 3.3	0.39
	24	**18.9 ± 0.5**	**0.7 ± 0.3**	**0.04**
	48	**2.24 ± 0.7**	**0.24 ± 0.05**	**0.012**
*Eh*prd	1	12.6 ± 5.8	6.1 ± 2.3	1.05
	3	**7.1 ± 2.46**	**2.5 ± 0.8**	**0.043**
	24	**4.35 ± 1.3**	**0.43 ± 0.24**	**0.05**
	48	1.9 ± 0.7	0.53 ± 0.3	0.26
*Eh*sod	1	0.88 ± 0.34	0.84 ± 0.4	1.5
	3	**6.3 ± 1.4**	**3.1 ± 1.2**	**0.032**
	24	**2.5 ± 1.1**	**0.81 ± 0.22**	**0.046**
	48	0.72 ± 0.4	0.32 ± 0.2	0.85

After human PCLS were *ex vivo* infected with *E*. *histolytica* or *E*. *dispar* trophozoites for different time spans (1, 3, 24 and 48 h), the relative quantification (RQ) was determined (by qPCR) for the mRNA of some genes obtained by a bibliographic analysis (S1 Table). (RQ) relative quantification, (SD) standard deviation, data expressed as the mean of 5 separate assays. Shown the RQ of some genes which codified to hypothetical proteins, the cardinal number added is only for personal identification. p value calculated by Student t-test.

## Discussion and conclusions

A key factor limiting the understanding of *Entamoeba* infection mechanisms, is the absence of an experimental model that can accurately reproduce the entire life cycle of these parasites. The establishment of such a model, should certainly help to clarify the mechanisms involved in parasite virulence and the host immune response, as well as environmental factors influencing the outcome of infection. Consequently, we sought to develop a reliable alternative model for studying the first events triggered by the cell-to-cell interaction between liver parenchyma and either *E*. *histolytica* or *E*. *dispar* trophozoites, employing human liver tissue explants (PCLS).

The PCLS preserved the structure and components of human liver tissue, with the hepatocytes in the typical sinusoidal settlement (hepatic stellate cells, hepatocytes and Kupffer cells).It was possible to identify some immune cells like lymphocytes and histiocytes. These findings coincide with other studies describing cell-to-cell as well as cell-to-extracellular matrix interactions. PCLS are metabolically competent, having active phase I and II drug metabolism enzymes during 24 h incubation periods [70–72]. The PCLS from hamsters also yielded intact liver tissue, and the *E*. *histolytica* infection showed similar characteristics[20,21].

Regarding the parasite-PCLS interaction, trophozoites decreased in number after 3 h of infection with *E*. *histolytica* or *E*. *dispar*. Whereas, it was still possible to retrieve live trophozoites after 24 h of *E*. *histolytica* infection, however, the trophozoites of *E*. *dispar* species continued to die until the end of the experiment. This agree with descriptions of amoebic liver abscess developed in hamsters and the PCLS of hamster liver infection [73, 20, 21]. It has been reported that in the liver of hamsters, trophozoites die during the first 6 h post-inoculation. After this period, however, ischemia due to inflammation generates an atmosphere of micro-anaerobiosis, a necessary condition for the survival of *E*. *Histolytica* trophozoites [74].

Tissue damage in PCLS maybe caused by the activation of virulence products of *E*. *histolytica* as well as from the host immune/inflammatory response triggered by the parasite, mostly during the first stages of the host-parasite relationship [74,75]. The contribution of host inflammation to tissue destruction has been kinetically demonstrated in various illustrative studies utilizing the experimental model of ALA induced in golden hamsters [14, 61,73].

The present results are also validated by the observation of a transient immune-inflammatory response in the PCLS from human tissue infected with the non-virulent *E*. *dispar* species. This same response was found when using the *in vivo* hamster model of ALA and the *ex-vivo* hamster model of PCLS infected with *E*. *histolytica* trophozoites [20,21,73]. The authors detected the formation of micro-abscesses after 12 h of cell-to-cell interaction.

There are some important similarities and differences between the current findings in PCLS from human tissue and previous data reported for hamster liver specimens. Regarding differences, the human liver parenchyma is more susceptible to changes in temperature and in O_2_/CO_2_ ratios during incubation periods. A major limitation of this experimental approach is the inability to standardize the postmortem time for obtaining the human liver specimens. We included specimens obtained from donors from 4–8 h postmortem, and proceeded to examine the reliability and reproducibility of the results. This variability in time for obtaining the liver sample does not exist with the use of hamster PCLS.

According to the current data, human liver explants can be viable if the organ specimens are obtained 4–8 h postmortem. On the other hand, viability was independent of the age or gender of the donor, or the cause of death (Table 1). Furthermore, binucleated hepatocytes were identified, providing a clear sign of cell viability and regeneration of liver tissue.

During the *E*. *histolytica* infection, the overexpressed genes were related to the host inflammatory response or the survival/virulence mechanisms of the parasite. Inflammation is a prerequisite for *E*. *histolytica* to survive in hepatic tissue, and colonization triggers the entire inflammatory process that enables the development of ALA [73,74,76]. There is increasing evidence that the presence of the parasite evokes an immune response characterized by the secretion of pro-inflammatory mediators, in which the intestinal epithelial cells act as antigen-presenting cells [76,77]. Histological analysis of human colonic biopsies has revealed slight infiltration of neutrophils, macrophages and dendritic cells into the submucosa at the very beginning of the ulceration process. However, an increase in the number of neutrophils, plasma cells, eosinophils, macrophages and T cells are found as the infection progresses [78].

Recent studies have provided evidence that chemokines and/or chemokine receptors can be crucial mediators for inflammation and tissue injury both in the intestine and the liver parenchyma. Chemokines are small molecules (8–11 kDa) capable of participate in immune and inflammatory responses, through chemoattraction and activation of leukocytes [64]. Whereas parasite-specific immune responses, regulated by cytokines and chemokines, modulate and drive the expression of the host defense, they may also contribute to infection-induced pathogenesis and the persistence of the parasite [79]. Whereas parasite-specific immune responses regulated by cytokines and chemokines, modulate and drive the expression of immunity, they may also contribute to infection-induced pathogenesis and the persistence of the parasite [74,80].

Since inflammation is necessary for the survival of *E*. *histolytica*, they possibly evade an effective immune response by modulating cytokine and chemokine production. It has been demonstrated that *E*. *histolytica* trophozoites increase the expression and secretion of chemokines and pro-inflammatory cytokines, including IL-1, IL-8, IL-6, GRO-a and GMCSF in stromal and epithelial cells [81,82]. These findings emphasize the active role of cytokines in amoeba-induced inflammation.

Moreover, in the present human model of infection, PCLS displayed immune cells (lymphocytes). Although, a characteristic cellular organization of an inflammatory response was not found, genes for proinflammatory cytokines (e.g., TNF α, IL-8 and INF-γ) were clearly overexpressed when the liver tissue was infected with *E*. *histolytica*. On the other hand, the *E*. *dispar* infection caused greater gene expression for an anti-inflammatory cytokine (IL-10). Unlike the current results, the expression of IL-10 has been reported in a hamster model during the initial stages of an infection with *E*. *histolytica*, followed by a sharp decrease in the level of this cytokine[21]. In the case of *E*. *dispar*, no information is available from that study. It is known that the inflammatory response is a prerequisite for parasite survival and for the adaptation of trophozoites for colonization of hepatic tissue, which leads to tissue damage. The current results regarding IL-10 and *E*. *dispar* suggest that one of the characteristics determining the non-pathogenic character of this species may be its inability to elicit a strong inflammatory response.

There is unambiguous evidence of an intense acute inflammatory reaction in hamsters during *E*. *histolytica* infection. During the initial stages of *E*. *histolytica* infection in the liver, viable trophozoites are in close contact with abundant polymorphonuclear cells and some eosinophils [20,73]. Tsutsumi et al. described the first events of liver abscess formation in hamsters after the inoculation of trophozoites in the liver parenchyma. Nevertheless, no evidence exists to demonstrate that this chronology of hamster liver infection events coincides with the process of human liver infection.

Concerning the protection of trophozoites from a hostile environment, certain metabolic genes, such as *Ehsod*, *Ehprd* and *Ehhsp-70*, were overexpressed with *E*. *histolytica* but not *E*. *dispar* infection. These enzymes have the capacity to protect parasites under certain conditions of oxidative and thermal stress [52–54], which indicates that *E*. *dispar* cannot adapt and survive in the hostile environment of liver parenchyma. Additionally, it has been shown that *E*. *histolityca* responds more strongly to oxidative stress than *E*. *dispar* and *E*. *histolytica* non-virulent Rahman strain, and surface localization of Prx *E*. *histolytica* is associated with virulence [83, 84].

Finally, the overexpression of genes linked to pathogenicity (*Ehcp-5*, *Ehcp-1*,*Eh*cp-2,*Ehlect* and *Ehap-a*) were presently detected in *E*. *histolytica-* but not *E*. *dispar*-infected tissue. It has been firmly established that the decisive action of these proteins induces an invasive process [75,76]. Although EhCp5 has proven to be more abundant thanCP1 and CP2, all these proteins are of a key importance to the invasive process in animal models [36, 37, 70]. The current results support the importance of EhCP in the virulence of *E*. *histolytica*.

In 2009, Bansal et al. observed that the low level of expression of the Gal/Nac lectin and amoebapore genes did not impede an *E*. *histolytica* invasion in the human *ex-vivo* intestinal model[22]. On the other hand, the expression of both proteins has been described in both human and hamster liver specimens in models of ALA. We herein show differences in the time of expression of amoebapores and the Gal/Nac lectin compared to the PCLS of hamster liver infected with *E*. *histolytica* trophozoites.

During an *E*. *histolytica* infection, as can be appreciated, there is simultaneous expression of genes related to the inflammatory response (*inf*-γ and *il*-8) and of pathogenicity genes (especially *Ehcrt*, *Ehcp-1*, *Ehcp-5*, *Ehcp2* and *EhLgl*), accompanied by the overexpression of genes encoding for enzymes that protect against oxidative and thermal stress (*Ehsod*, *Ehprd* and *Ehhsp-70*). Accordingly, in the initial stages of the host-parasite interaction, these factors give rise to the adaptation and survival of the parasite followed by the onset of the invasive process.

In conclusion, the use of an *ex vivo* PCLS model with human tissue is a suitable alternative for analyzing an *E*. *histolytica* infection to determine the simultaneous kinetics of expression of genes associated with parasite survival and its pathogenicity, as well as those related to the host inflammatory response. The interaction between *E*. *histolytica* trophozoites and PCLS from human liver tissue seems to reproduce the early impact of the parasite on its human host.

## Supporting information

S1 Table List of selected genes by bibliographic analysis, for expression assays by qPCR in the PCLS human liver model. (XLSX)

S1 Fig Densitometric analysis of immune-histochemical assays. (XLSX)
