# Correction: Differential expression of pathogenic genes of *Entamoeba histolytica* vs *E*. *dispar* in a model of infection using human liver tissue explants

**DOI:** 10.1371/journal.pone.0210895

**Published:** 2019-01-10

**Authors:** Cecilia Ximénez, Enrique González, Miriam Nieves, Ulises Magaña, Patricia Morán, Marco Gudiño-Zayas, Oswaldo Partida, Eric Hernández, Liliana Rojas-Velázquez, Ma. Carmen García de León, Héctor Maldonado

Table 4 is incorrect. Please see the correct Table 4 here.

**Table 4:** Expression levels of genes linked to mechanisms of pathogenesis in the ex vivo infection model, selected by bibliographic revision.

CLAVE	Time (h)	RQ ± SD		p value
		*E*. *histolytica*	*E*. *dispar*	
**Ehoxired**	1	6049 ± 1527	3822 ± 1231	0.072
	3	2320 ± 127.3	820.5 ± 269	0.045
	24	592.2 ± 125	75 ± 25	0.033
	48	21 ± 12.5	0.8 ± 0.23	0.025
**Ehaig-1**	1	80.3 ± 32.3	16.3 ± 2.6	0.04
	3	361.1 ± 72.4	0.64 ± 0.4	0.03
	24	4154. 4 ± 201	0.21 ± 0.1	0.02
	48	3.4 ± 1.2	0.96 ± 0.4	0.017
**Eh hsp-20kDa**	1	957.2 ± 191	27.4 ± 6.6	0.036
	3	1236.8 ± 327	39.2 ± 6.1	0.023
	24	29.3 ± 5.6	9.5 ± 6.5	0.03
	48	35.8 ± 9.1	0.05 ± 0.0006	0.04
**Ehpeptidase**	1	2.1 ± 0.6	0.044 ± 0.014	0.015
	3	8.25 ± 1.7	0.66 ± 0.25	0.026
	24	2.7± 1.9	0.43 ± 0.12	0.045
	48	2.23 ± 0.23	0.05 ± 0.03	0.04
**Eh20kDa**	1	0.37 ± 0.1	0.66 ± 0.4	0.93
	3	70.7 ± 28	0.3 ± 0.2	0.042
	24	4.1 ± 0.33	0.71 ± 0.4	0.015
	48	2.3 ± 0.3	0.55 ± 0.4	0.14
**Eh dovasa**	1	0.41 ± 0.03	0.9 ± 05	0.18
	3	2.1 ± 0.65	0.51 ± 0.2	0.032
	24	0.9 ± 0.3	0.15 ± 0.01	0.046
	48	0.9 ± 0.14	0.12 ± 0.043	0.042
**Ehthiored**	1	0.31 ± 0.023	0.31 ± 0.023	0.083
	3	0.22 ± 0.1	0.13 ± 0.05	0.067
	24	0.5 ± 0.03	0.2 ± 0.1	0.042
	48	0.7 ± 0.14	0.2 ± 0.1	0.03
**Ehhypo1**	1	69 ± 32	0.23 ± 0.1	0.032
	3	44.4 ± 1.3	0.9 ± 0.2	0.03
	24	61.4 ± 25	0.53 ± 0.14	0.02
	48	40 ± 15.4	0.5 ± 0.1	0.02
**Ehhypo3**	1	1.02 ± 0.4	1.7 ± 0.001	0.125
	3	0.21 ± 0.1	13.2 ± 4.7	0.031
	24	0.98 ± 0.7	7.4 ± 0.01	0.046
	48	0.22 ± 0.01	0.76 ± 0.5	0.062
**Ehhypo4**	1	34.82± 0.4	5.4 ± 2.3	0.017
	3	8.73 ± 2.5	6.2 ± 1	0.82
	24	18.3 ± 6.1	17.1 ± 5	0.062
	48	15.1 ± 6	0.53 ± 0.3	0.032
**Ehhypo5**	1	191.6 ±71.5	914.9 ± 95.5	0.15
	3	1053 ± 33.6	3.6 ± 2.2	0.017
	24	3.9 ±1.3	0.27 ± 0.1	0.033
	48	0.75 ± 0.31	0 .63 ± 0.4	0.072
**Ehhypo6**	1	73.01 ± 32	0.01 ± 0.001	0.034
	3	145.1 ± 63	0.034 ± 0.014	0.021
	24	2.6 ± 0.7	0.02 ± 0.005	0.045
	48	1.71 ± 0.7	0.01 ± 0.01	0.043
**Ehhypo7**	1	0.00011 ± 0.00006	0.0083 ± 0.0006	0.45
	3	3.7 ± 0.85	0.48 ± 0.05	0.29
	24	286.1 ± 57.1	0.87 ± 0.45	0.017
	48	2.1 ± 0.45	0.15 ± 0.05	0.124
**Ehypo8**	1	6.4 ± 2.6	3.2 ± 0.5	0.083
	3	13.6 ± 0.43	4.8 ± 1.5	0.02
	24	2.83 ± 0.5	1.3 ± 0.23	0.31
	48	0.95 ± 0.3	0.31 ± 0.02	0.073
**Ehhypo9**	1	3.1 ± 1.2	8.13 ± 0.95	0.02
	3	0.56 ± 0.29	0.56 ± 0.4	0.03
	24	129.4 ± 27.6	0.18 ± 0.1	0.025
	48	2.2 ± 1.1	0.44 ± 0.04	0.04
**Ehhypo10**	1	0.74 ± 0.3	0.05 ± 0.013	0.097
	3	0.012 ± 0.006	0.045 ± 0.004	0.94
	24	0.041 ± 0.003	0.01 ± 0.004	0.31
	48	0.05 ± 0.01	0.00023 ± 0.0001	0.88

After human PCLS were ex vivo infected with *E*. *histolytica* or *E*. *dispar* trophozoites for different time spans (1, 3, 24 and 48 h), the relative quantification (RQ) was determined (by qPCR) for the mRNA of some genes obtained by a bibliographic analysis. (RQ) relative quantification, (SD) standard deviation, data expressed as the mean of 5 separate assays. Shown the RQ of some genes which codified to hypothetical proteins, the cardinal number added is only for personal identification. p value calculated by Student t-test.
